# Knowledge and Attitudes of Dental Students on E-portfolios as a Tool for Learning, Assessment, and Professional Development in Dental Education

**DOI:** 10.7759/cureus.81980

**Published:** 2025-04-09

**Authors:** Hung T Dang, Tuan A Tran, Ngoc N Vo Truong, Hang M Luong, Hoan Q Nguyen

**Affiliations:** 1 Oral and Maxillofacial Surgery, School of Dentistry, Hanoi Medical University, Hanoi, VNM; 2 Dentistry, School of Dentistry, Hanoi Medical University, Hanoi, VNM; 3 Pediatric Dentistry, School of Dentistry, Hanoi Medical University, Hanoi, VNM

**Keywords:** assessment, e-portfolios, learning, professional development, tool

## Abstract

Background

E-portfolios are evolving electronic/online resources that record, store, and archive the artifacts of learning and reflection for an individual learner. They have become more popular nowadays. This study aimed to evaluate the knowledge and attitudes of dental students regarding the use of e-portfolios as a tool for assessment, learning, and professional development in dental education.

Methods

Three hundred seven dental students completed a five-point Likert scale questionnaire, which was administered using Google Forms (Google, Inc., Mountain View, CA) on e-portfolios. The questionnaire comprised three sections: general information, knowledge, and attitude assessments. Data were stored in EpiData 3.1 (Jens M. Lauritsen, Odense, Denmark) and analyzed with Stata 16.0 (StataCorp LLC, College Station, TX).

Results

The research shows that 83.7% of students appreciate using portfolios for reflecting on their skills, and 82.1% believe that e-portfolios are effective for self-assessment. Portfolios help 55.0% of students identify their strengths and weaknesses, and 68.7% report experiencing better independent learning. Moreover, 70.0% of students feel that portfolios help to connect theoretical knowledge with practical application. Challenges include 71.3% of students finding portfolios time-consuming and 49.5% feeling stressed. It is essential to have effective guidance from instructors, with 77.2% agreeing on the importance of clear goals and 70.7% valuing faculty consultations.

Conclusion

While e-portfolios are generally viewed positively by the majority of dental students for their role in learning, assessment, and professional development, the study highlights the need for proper guidance and integration into the overall grading system to address concerns about time and stress.

## Introduction

After graduating, dental professionals become direct providers of patient care without the guidance of faculty. Learning to assess their own skills prepares graduates to succeed in their careers by adapting to changes in the work environment. It is essential for them to identify gaps in their knowledge and skills and determine how to fill them in order to practice evidence-based clinical care and engage in lifelong learning [1].

E-portfolios, or electronic portfolios, were first introduced in the 1990s and became more popular in the education sector in the early 2000s [2]. It was first presented as paper portfolios and now has transferred into web-based platforms in many types, such as assessment e-portfolios, showcase e-portfolios, reflective or learning e-portfolios, professional e-portfolios, and academic e-portfolios, which enable learners to arrange and distribute a wide range of digital documents, including text, images, and videos [3], and this change has provided increased flexibility in storing and accessing documents [4].

E-portfolios are defined by scholars as tools for documenting both learning outcomes and learning processes [5]. Barrett (2005) highlights their ability to gather and organize materials in various forms [6], while Meyer (2010) describes e-portfolios as digital archives for education and assessment, where lecturers assess learners' assignments, projects, and reflections. It supports formative feedback during learning and summative evaluation at the end of a course [7]. In addition to storing and organizing materials, e-portfolios also support self-assessment, feedback, and personal development. They can record the learning process, achievements, and development of learners [8]. Lorenzo and Ittelson define e-portfolios as electronic collections of knowledge, resources, and achievements of an individual, group, or organization [9]. Overall, e-portfolios are convenient tools for documenting the personal experiences of both teachers and learners, which are useful for assessing the learning and working process [10].

The development of educational technology was followed by the rise of e-portfolios in education. Learners and educators recognize that technology has the potential to develop education, and e-portfolios are now considered a valuable tool for Personalized Learning Environments (PLEs) [7]. E-portfolios improve the learning experience by giving learners more autonomy. They assist learners in establishing goals, identifying learning strategies, and developing self-assessment skills [7,11,12]. It can also help learners increase their feeling of responsibility, critical thinking skills, and learning mindsets. Whether in paper or electronic form, e-portfolios allow educators to monitor and evaluate students' progress during a course or subject from multiple perspectives [13]. E-portfolios are visual records of work in any profession, offering useful data for analysis and evaluation [14].

With its increasing worldwide adoption, particularly in higher education, e-portfolios are increasingly being integrated into teaching, learning, assessment, instruction, and professional development practices [3,5]. Although there have been numerous studies on the usefulness of e-portfolios, research on learners' knowledge and attitudes toward e-portfolios is limited. Furthermore, this is the first time an e-portfolio has been implemented in a new curriculum at Hanoi Medical University's School of Dentistry. As a result, to fill those gaps, we performed this study aimed to evaluate the knowledge and attitudes of dental students in the use of e-portfolios as a tool for assessment, learning, and professional development in dental education.

## Materials and methods

This cross-sectional study was conducted on 307 dental students learning at a medical university in Vietnam from January 2024 to June 2024.

Research question

A primary research question was formulated for the research: “Evaluate knowledge and attitudes of dental students regarding the use of e-portfolios as an assessment, learning, and professional development tool in dental education.”

Also, the following PEO criteria were defined: Population is dental students; Exposure is e-portfolios; and Outcomes are knowledge and attitudes.

Eligibility criteria

The participant must meet the following criteria to be considered for evaluation. The participant must be a clinical year dental student who has used e-portfolios for at least one semester. The validity of the participants must be confirmed by the Head of the Training Management Department.

Participants who met only some of the inclusion criteria were not considered. Moreover, the exclusion criteria were as follows. Lecturers confirmed that the participants did not meet the criteria for sufficient mental and physical health based on the International Classification of Diseases (ICD) [15], and participants did not agree to participate in the study.

Data collection

Participants who met the eligibility criteria were given a questionnaire about e-portfolios to complete from January 2024 to June 2024 via Google Form (Google, Inc., Mountain View, CA). They were informed about the aim and purpose of the study, provided with an explanation of the questionnaire, and then invited to participate in the study.

The e-portfolio used in the research was developed by the university's Training Management Department. It serves four main functions: (1) showcase the core values of the subject, (2) highlight the practice targets, (3) provide a record of practice, and (4) include student comments. Students were obligated and trained to use the e-portfolio as a part of the assessment scheme starting in the second half of the second semester of their first year.

Questionnaire

**Figure 1:**
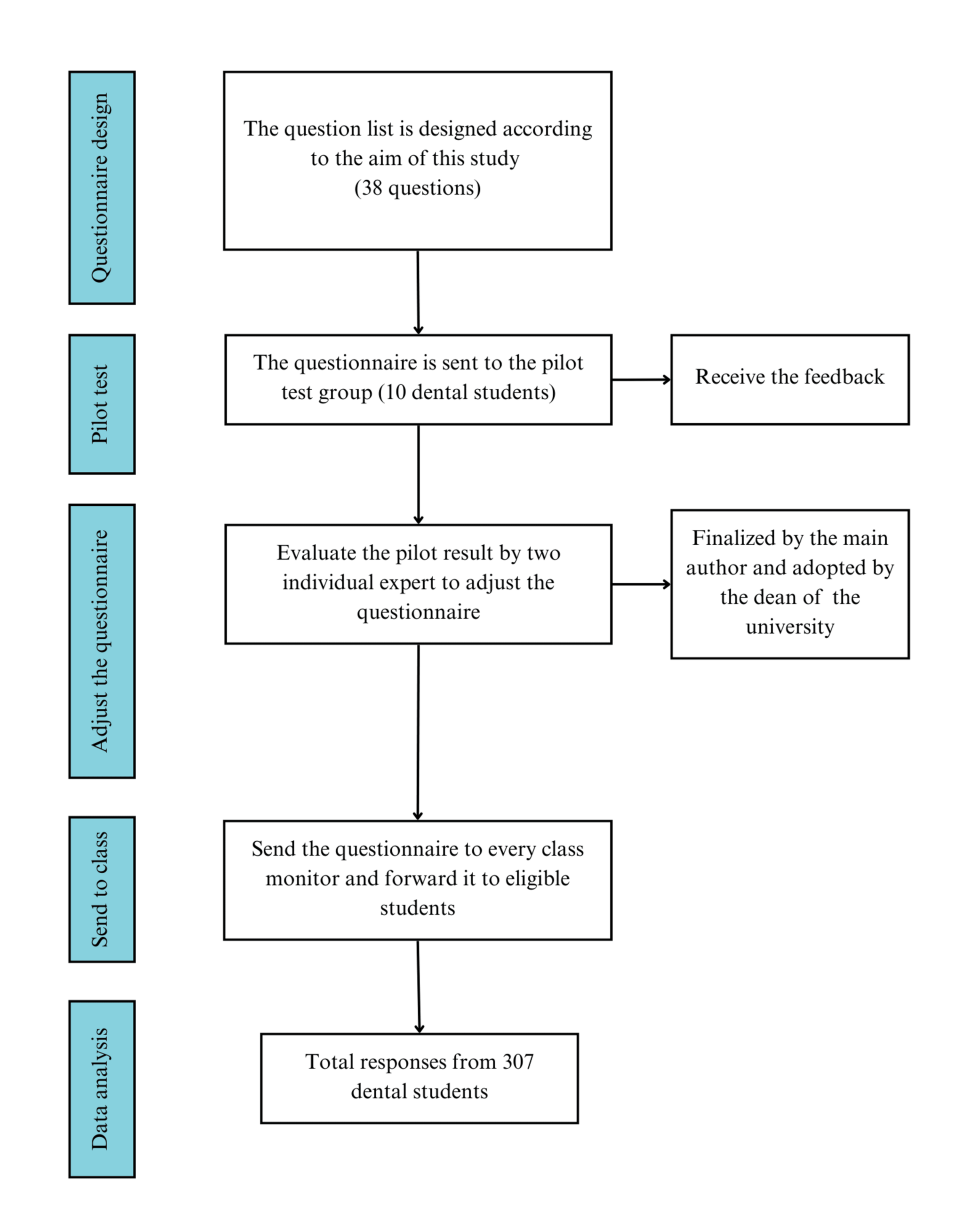
Questionnaire establishment flowchart

The five-point Likert scale questionnaire was developed based on the research of Devarajoo et al. [16], Crisol Moya et al. [17], and Sanchez et al. [14]. It was translated into Vietnamese and was pre-tested by a group of 10 dental students. The result of the pilot test was then evaluated by two individual experts, who adjusted the questionnaire to improve the clarification according to the feedback from this group before being finalized by the main author and adopted by the dean of the university (Figure 1).

The final version used in our study comprised three sections, including one open-ended and 37 closed-ended questions. The first section gathered general information, including sex, year, and age. The second section contained questions to assess dental students' general knowledge of e-portfolios. The third section contained three parts to assess the attitude of dental students about the applications of e-portfolios in personal and professional development, their feedback on using e-portfolios, and the lecturer's support and guidance in using e-portfolios. The final questionnaire was then transferred to an online format and distributed to eligible participants for data collection purposes, and the participants will receive a gift from the researchers. The descriptive questionnaire used in the study is presented in the Appendices.

Data analysis

The data were stored using EpiData version 3.1 (Jens M. Lauritsen, Odense, Denmark), and statistical analysis was conducted using Stata version 16.0 (StataCorp LLC, College Station, TX), and the descriptive analyses were expressed as numbers (N) and percentages (%).

## Results

Demographic characteristics

The students who were interviewed for this study were starting their studies in the second year because most first-year students had not used portfolios. The majority of participants were fifth-year students (80 (26.1%)), followed by second-year (69 (22.5%)) and fourth-year students (66 (21.5%)). For the gender ratio in the student group, males accounted for 132 (43.0%), and females accounted for 175 (57.0%). The age of the students was mainly concentrated between 20 and 23 years old, with students at the age of 23 representing the highest ratio at 73 (23.8%) (Table 1).

**Table 1:** Demographic characteristics (N = 307)

Characteristics		N (number)	% (proportion)
Academic year	First year	0	0.0
	Second year	69	22.5
	Third year	60	19.5
	Fourth year	66	21.5
	Fifth year	80	26.1
	Sixth year	32	10.4
Sex	Male	132	43.0
	Female	175	57.0
Age	20	60	19.5
	21	65	21.2
	22	67	21.8
	23	73	23.8
	24	39	12.7
	25	3	1.0

Knowledge of dental students about e-portfolios

The majority of students either agreed or strongly agreed with the benefits of the portfolio. They considered it to be a useful tool for reflecting on learning (257 (83.7%)) and a safe place to store evidence of skills and knowledge (256 (83.4%)). Additionally, students largely perceived the e-portfolio as an electronic version of a paper portfolio (268 (87.3%)) and an important tool for self-assessment (252 (82.1%)) (Table 2). 

**Table 2:** General knowledge of dental students about e-portfolios

Characteristics	Strongly agree/agree	Consider	Strongly disagree/disagree
The portfolio serves as evidence of skills and provides an opportunity to showcase the learning process and reflect on what we are learning.	257 (83.7%)	41 (13.4%)	9 (2.9%)
The portfolio is a place for us to gain a clearer understanding of our learning plan - where I started and where I am headed.	237 (77.2%)	60 (19.5%)	10 (3.3%)
This is a secure electronic repository for me to gather and store evidence of the skills and knowledge I have acquired.	256 (83.4%)	38 (12.4%)	13 (4.2%)
It's a digital repository containing samples of the coursework I completed.	269 (87.6%)	29 (9.5%)	9 (2.9%)
E-portfolio is an electronic version of a paper portfolio.	268 (87.3%)	34 (11.1%)	5 (1.6%)
It is a digital tool that enables self-assessment and allows me to document my experiences throughout the course.	252 (82.1%)	47 (15.3%)	8 (2.6%)

Applications of e-portfolio in personal and professional development

Portfolios play a significant role in helping students become more aware of their strengths (169 (55.0%)) and weaknesses (169 (55.0%)). However, 112 (36.5%) of students consider their awareness of weaknesses. Students believe that portfolios develop their ability to learn independently (211 (68.7%)) and emphasize the value of continuous learning (232 (75.6%)). Additionally, a high percentage of students (223 (72.6%)) think that portfolios help develop responsibility for their own professional development (Table 3).

**Table 3:** Applications of e-portfolio in personal and professional development

Characteristics	Strongly agree/agree	Consider	Strongly disagree/ disagree
Personal development			
My portfolio has increased my awareness of my strengths.	169 (55.0%)	108 (35.2%)	30 (9.8%)
My portfolio has increased my awareness of my weaknesses.	169 (55.0%)	112 (36.5%)	26 (8.5%)
My portfolio has helped me develop my ability to learn independently.	211 (68.7%)	78 (25.4%)	18 (5.9%)
My portfolio has helped me value continuous learning.	232 (75.6%)	62 (20.2%)	13 (4.2%)
My portfolio has helped me improve my self-esteem and sense of self-worth.	217 (70.7%)	73 (23.8%)	17 (5.5%)
My portfolio has improved my self-confidence.	158 (51.5%)	118 (38.4%)	31 (10.1%)
My portfolio has helped me develop responsibility for my own professional development.	223 (72.6%)	69 (22.5%)	15 (4.9%)
Professional development			
My e-portfolio helps me improve my ability to connect theory to practice.	215 (70.0%)	73 (23.8%)	19 (6.2%)
My e-portfolio helps me identify the areas where I have good knowledge.	211 (68.7%)	77 (25.1%)	19 (6.2%)
My e-portfolio helps me identify the areas where I have good skills.	214 (69.7%)	67 (21.8%)	26 (8.5%)
My e-portfolio helps me identify the areas where I lack sufficient knowledge.	209 (68.1%)	80 (26.1%)	18 (5.9%)
My e-portfolio helps me identify the areas where I lack good skills.	209 (68.1%)	76 (24.8%)	22 (7.2%)
My e-portfolio helps me promote critical thinking.	189 (61.6%)	88 (28.7%)	30 (9.8%)
My e-portfolio helps me improve my reflexive skills.	172 (56.0%)	103 (33.6%)	32 (10.4%)

Students believe that e-portfolios also play a crucial role in helping them improve their ability to connect theory to practice (215 (70%)) and in identifying skills (214 (69.7%)) and knowledge (209 (68.1%)) gaps. Additionally, e-portfolios can promote learners’ critical thinking (189 (61.6%)) and reflective skills (172 (56.0%)). However, 10.0-20.0% of students remain confused about whether portfolios truly help them identify areas for improvement in their skill practice (Table 3).

General student feedback on using e-portfolios

The majority of students (219 (71.3%)) agreed that creating a portfolio takes a significant amount of time, and nearly half (152 (49.5%)) found it to be stressful. Most students (235 (76.6%)) expressed a desire to use a portfolio as a tool for learning, and 207 (67.4%) saw it as an assessment tool. However, 118 (38.4%) felt that e-portfolios would add value to learning if it does not contribute to the final grade (Table 4).

**Table 4:** General student feedback on using e-portfolios

Characteristics	Strongly agree/agree	Consider	Strongly disagree/disagree
It takes a long time to complete an e-portfolio.	219 (71.3%)	69 (22.5%)	19 (6.2%)
Using an e-portfolio is stressful.	152 (49.5%)	109 (35.5%)	46 (15.0%)
I possess good clinical writing skills.	149 (48.5%)	129 (42.0%)	29 (9.5%)
I want an e-portfolio to be a tool for evaluating the knowledge and skills I have.	207 (67.4%)	81 (26.4%)	19 (6.2%)
I want an e-portfolio to be a tool for learning.	235 (76.6%)	59 (19.2%)	13 (4.2%)
An e-portfolio will only add value to learning if the portfolio score contributes to the final grade.	166 (54.1%)	99 (32.2%)	42 (13.7%)
An e-portfolio will add value to learning if it does NOT contribute to the final grade.	118 (38.4%)	134 (43.7%)	55 (17.9%)
Assessing an e-portfolio both in aggregate and separately increases its learning value.	206 (67.1%)	91 (29.6%)	10 (3.3%)
I will have difficulty completing an e-portfolio if it is used for grading.	134 (43.7%)	109 (35.5%)	64 (20.8%)
I will have difficulty completing an e-portfolio if it is used for individual assessment and not for grading.	121 (39.4%)	119 (38.8%)	67 (21.8%)

Evaluation of lecturers' support and guidance in using e-portfolios

Students appreciated the clarity of the e-portfolio's objectives (237 (77.2%)) and felt they received adequate support from lecturers through feedback (217 (70.7%)). However, around 72 (23.5%) of students are still confused about how to use the e-portfolios (Table 5).

**Table 5:** Evaluation of lecturers' support and guidance in using e-portfolios

Characteristics	Strongly agree/agree	Consider	Strongly disagree/disagree
E-portfolio objectives are clear.	237 (77.2%)	52 (16.9%)	18 (5.9%)
I know what evidence of learning is needed in my e-portfolio.	198 (64.5%)	88 (28.7%)	21 (6.8%)
I know how to use my e-portfolio.	217 (70.7%)	72 (23.5%)	18 (5.8%)
There are frequent feedback sessions from lecturers on e-portfolio development.	186 (60.6%)	83 (27.0%)	38 (12.4%)
Lecturers' feedback is helpful.	217 (70.7%)	71 (23.1%)	19 (6.2%)

## Discussion

E-portfolios play a crucial role in helping students systematically track and manage their learning process. According to our survey, most of the students believe that e-portfolios support them in reflecting on and understanding their learning progress. This self-evaluation and reflection process is beneficial for improving students' critical thinking skills and organizing their knowledge. Previous studies by Barrett in 2005 and Meyer in 2010 also support the idea that e-portfolios are not just storage spaces for documents but also tools for self-reflection and monitoring of the learning process [6,7]. Additionally, e-portfolios assist students in setting their learning goals and planning their long-term career paths [7,11,12]. However, some students encounter challenges in using e-portfolios to synthesize knowledge, particularly when they lack sufficient guidance from their lecturers. Moreover, a large number of students find e-portfolios time-consuming, especially when balancing academic and clinical activities. This finding is consistent with the results of Devarajoo’s study in 2020 [16]. Students who are not familiar with the tool often struggle to organize their materials, leading to feelings of stress and pressure [17]. The lack of specific guidance or support from instructors further compounds the challenge, turning the e-portfolio into a burden rather than an effective support tool [16-18].

In our study, the majority of students found e-portfolios to be effective for self-assessment, helping them identify their strengths and weaknesses. This finding aligns with previous studies, which have highlighted e-portfolios' ability to aid students in self-assessment and skill development [7,11,12]. This is especially important in the medical industry, as students must constantly refine their clinical skills. Self-assessment also assists students in developing strategies to improve their deficiencies and professional skills [19-22]. However, this procedure might be difficult without regular input from instructors, reducing the usefulness of e-portfolios [16,18-23]. While e-portfolios support self-assessment, the participants still found that it was stressful when using this tool. Our study suggests that this pressure stems from the need to constantly update and evaluate one's learning process, alongside meeting specific standards for e-portfolio completion [17,24]. Lack of guidance and support from lecturers may leave students confused about the use and the accuracy of reflecting on their skill development [16]. Furthermore, self-assessment demands proficient writing and reflection skills, which not all students possess.

Most of the students reported that e-portfolios help them in the connection of theoretical knowledge with clinical practice, a crucial aspect in the dentistry field where clinical skills are important. Moreover, a large number of students indicated that e-portfolios enhanced their capacity for independent learning, thereby creating the skills that are essential for lifelong learning. These findings can be found in Klenowski's research in 2006 and 2010 [25,26], which emphasized the important role of e-portfolios in developing learners' study skills and enabling them to plan and monitor their career development. By storing documents, skills, and accomplishments [27-29], e-portfolios offer students a tool for showcasing their credentials to prospective employers and professional organizations [30]. While e-portfolios are instrumental for professional development, a significant proportion of students perceive their completion as truly valuable only if integrated into the formal assessment system [16,17]. Indeed, students still believe that e-portfolios would yield greater educational value if their scores contributed to the final grade. This viewpoint suggests that without formal integration, many students may lack the motivation to fully engage with e-portfolios [17,18]. Furthermore, the lack of alignment between e-portfolios and the present assessment framework diminishes their efficacy.

The study revealed that most of the students believe that the purpose of using e-portfolios should be clearly explained, and that the important role of receiving guidance from their lecturers should be emphasized. This can be found in the research of Devarajoo et al., which concluded that e-portfolios cannot reach the core value of using e-portfolios without lecturer support [16]. The comprehensive guidance and crucial feedback assist students in comprehending the utilization of e-portfolios for their personal development. Lecturer support plays a significant role in contributing to the reduction of student stress when using e-portfolios [24] and facilitating optimal usage of the tool [16]. Despite the support of lecturers, a significant number of students expressed their confusion regarding the usage of e-portfolios [16]. This might be due to inadequate instruction or unimportant information provided by their lecturers, which might be a challenge for students in understanding and using the e-portfolio effectively. Furthermore, the lack of regular feedback from lecturers can be a challenge for students to evaluate and adjust their learning process [16]. These results emphasized the need for the improvement of the guidance from the lecturers in supporting learners to understand the core values and true usage of e-portfolios.

Some important limitations of this study should be acknowledged to guide future research more effectively. Despite the restriction of invalid data resulting from patients' commitments, there were still several instances of low-quality data due to the participants' attitudes. These attitudes were influenced by their varying levels of knowledge, experience, and needs. Future research should focus on improving students' attitudes toward completing the questionnaire before distributing it to each dental student. Another limitation of this study is that it only uses descriptive statistics, which give a broad picture of the data but don't allow for deeper analysis or comparisons between different groups. This means we can't make stronger conclusions or identify any significant patterns. Using more advanced statistical methods in future studies could help provide a clearer, more detailed understanding of the findings.

Additionally, not the entire class agreed to participate in the study, which was a result of the eligibility criteria and the attitudes of participants while completing the online questionnaire. Future research should consider providing participants with an offline questionnaire to achieve a larger sample size.

## Conclusions

This study found that the majority of dental students believe e-portfolios can offer numerous benefits for learning, assessment, and professional development by enhancing key aspects. However, challenges remain in their use. To fully realize the advantages of e-portfolios, dental educators should provide proper guidance and regular feedback and integrate them into the grading system. Future studies should also explore advanced statistical methods and consider demographic variables to gain deeper insights into their impact.

## Appendices

Descriptive questionnaire used in the study

I. Demographic characteristics

1. Which academic year are you in?

A. 1st year

B. 2nd year

C. 3rd year

D. 4th year

E. 5th year

F. 6th year

2. What is your biological gender?

A. Male

B. Female

3. How old are you?

A. 20

B. 21

C. 22

D. 23

E. 24

F. 25

II. General knowledge of dental students about e-portfolios

Answer from 1 - strongly agree to 5 - strongly disagree

4. The portfolio serves as evidence of skills and provides an opportunity to showcase the learning process and reflect on what we are learning.

5. The Portfolio is a place for us to gain a clearer understanding of our learning plan - where I started and where I am headed.

6. This is a secure electronic repository for me to gather and store evidence of the skills and knowledge I have acquired.

7. It's a digital repository containing samples of the coursework I completed.

8. E-portfolio is an electronic version of a paper portfolio.

9. It is a digital tool that enables self-assessment and allows me to document my experiences throughout the course.

III. Applications of e-portfolio in personal and professional development

Answer from 1 - strongly agree to 5 - strongly disagree

10. My portfolio has increased my awareness of my strengths.

11. My portfolio has increased my awareness of my weaknesses.

12. My portfolio has helped me develop my ability to learn independently.

13. My portfolio has helped me value continuous learning.

14. My portfolio has helped me improve my self-esteem and sense of self-worth.

15. My portfolio has improved my self-confidence.

16. My portfolio has helped me develop responsibility for my own professional development.

17. My e-portfolio helps me improve my ability to connect theory to practice.

18. My e-portfolio helps me identify the areas where I have good knowledge.

19. My e-portfolio helps me identify the areas where I have good skills.

20. My e-portfolio helps me identify the areas where I lack sufficient knowledge.

21. My e-portfolio helps me identify the areas where I lack good skills.

22. My e-portfolio helps me promote critical thinking.

23. My e-portfolio helps me improve my reflexive skills.

IV. General student feedback on using e-portfolios

Answer from 1 - strongly agree to 5 - strongly disagree

24. It takes a long time to complete an e-portfolio.

25. Using an e-portfolio is stressful.

26. I possess good clinical writing skills.

27. I want an e-portfolio to be a tool for evaluating the knowledge and skills I have.

28. I want an e-portfolio to be a tool for learning.

29. An e-portfolio will only add value to learning if the portfolio score contributes to the final grade.

30. An e-portfolio will add value to learning if it does NOT contribute to the final grade.

31. Assessing an e-portfolio both in aggregate and separately increases its learning value.

32. I will have difficulty completing an e-portfolio if it is used for grading.

33. I will have difficulty completing an e-portfolio if it is used for individual assessment and not for grading.

V. Evaluation of lecturers' support and guidance in using e-portfolios

Answer from 1 - strongly agree to 5 - strongly disagree

34. E-portfolio objectives are clear.

35. I know what evidence of learning is needed in my e-portfolio.

36. I know how to use my e-portfolio.

37. There are frequent feedback sessions from lecturers on e-portfolio development.

38. Lecturers' feedback is helpful.
